# Delayed Recognition of Autism Spectrum Disorder and Off-Label Lurasidone After Intolerance to Risperidone and Aripiprazole in a Latin American Child: A Case Report

**DOI:** 10.7759/cureus.117158

**Published:** 2026-09-29

**Authors:** Leiby Orellana Banegas, Raul Urbina, Yahaira Iveth I Flores Antúnez, Jessy A Mejia

**Affiliations:** 1 General Medicine, Universidad Católica de Honduras Nuestra Señora Reina de la Paz, San Pedro Sula, HND; 2 Pediatrics, Universidad Católica de Honduras Nuestra Señora Reina de la Paz, San Pedro Sula, HND

**Keywords:** autism spectrum disorder (asd), irritability, latin american, lurasidone, obsessive-compulsive disorder, off-label treatment, pediatric psychiatry

## Abstract

Autism spectrum disorder (ASD) can be difficult to recognize when co-occurring psychiatric symptoms are more clinically prominent, which may delay diagnosis. We report the case of a nine-year-old Latin American boy whose ASD and obsessive-compulsive disorder (OCD) were not diagnosed until approximately 27 months after initial presentation with irritability, mood lability, anxiety, and, subsequently, hallucinatory experiences; these symptoms were initially managed as primary psychiatric conditions. During this interval, fluoxetine was discontinued due to symptom worsening, sertraline produced only partial benefit, and both Food and Drug Administration (FDA)-approved agents for irritability associated with autistic disorder were discontinued because of adverse effects: risperidone due to significant weight gain and aripiprazole due to nausea, vomiting, and dizziness. A detailed neurodevelopmental reassessment subsequently identified longstanding social communication difficulties, rigid routines, sensory sensitivities, and restricted interests, supporting diagnoses of ASD and OCD. Low-dose lurasidone (20 mg nightly) was initiated off-label alongside continued sertraline. Over the following year, the patient demonstrated sustained improvement in irritability, aggression, self-injurious behavior, anxiety, and sleep disturbance. Hallucinatory experiences resolved within approximately three months and did not recur, with no significant adverse effects observed. This case illustrates how more prominent psychiatric symptoms can mask ASD and describes the individualized use of off-label lurasidone after intolerance to approved agents. However, the temporal association with clinical improvement does not establish causality, and findings from a single case cannot demonstrate efficacy.

## Introduction

Autism spectrum disorder (ASD) is a neurodevelopmental condition characterized by persistent difficulties in social communication and interaction, together with restricted or repetitive patterns of behavior, interests, or activities [1]. Psychiatric comorbidity is common in autistic individuals; a large systematic review and meta-analysis found substantial rates of co-occurring attention-deficit/hyperactivity disorder, anxiety disorders, depressive disorders, and obsessive-compulsive disorder (OCD) [2]. These co-occurring conditions may contribute substantially to functional impairment and, when clinically prominent, can obscure the underlying neurodevelopmental presentation and delay recognition of ASD.

Distinguishing OCD from the repetitive behaviors intrinsic to ASD can be diagnostically challenging. OCD is typically characterized by intrusive, unwanted thoughts and compulsions performed to reduce distress. In contrast, ASD-related repetitive behaviors are often ego-syntonic, serve an emotion-regulating function, or are experienced as part of the individual's identity. These presentations can overlap phenomenologically, so a detailed longitudinal history is often required to determine whether both conditions are present [3].

Psychotic-like or hallucinatory experiences create an additional diagnostic challenge. Individuals with ASD may report perceptual experiences, unusual beliefs, internal dialogue, sensory phenomena, or imaginative experiences that can be difficult to classify using frameworks developed for primary psychotic disorders. A meta-analysis has suggested that psychotic experiences are reported more frequently in ASD than in the general population and that careful assessment of developmental level, language, insight, distress, persistence, and functional consequences is necessary [4]. A recent clinical review similarly emphasizes that hallucinations, delusions, and negative symptoms often present in attenuated or atypical forms in ASD and that limited verbal communication can make them especially difficult to elicit and classify, which has immediate implications for treatment, prognosis, and access to care [5]. For this reason, we use the descriptive term "hallucinatory symptoms" in this report rather than assuming a primary psychotic disorder.

For severe irritability associated with autistic disorder, risperidone and aripiprazole are the two agents with US Food and Drug Administration (FDA) approval. Both can be effective, but pediatric treatment is frequently constrained by adverse effects, including weight and metabolic changes, prolactin elevation, sedation, activation, akathisia, and gastrointestinal symptoms [6,7]. When these treatments are not tolerated, clinicians may consider off-label alternatives on an individualized basis after weighing the limited evidence, symptom targets, previous adverse effects, drug interactions, and monitoring capacity [8].

Lurasidone is a second-generation antipsychotic with dopamine D2 and serotonin 5-HT2A antagonism, 5-HT7 antagonism, and partial agonism at 5-HT1A receptors [8]. In the United States, it is approved for schizophrenia in adolescents aged 13-17 years and for bipolar depression in children and adolescents aged 10-17 years; its use for ASD-associated irritability is off-label [9]. Lurasidone is contraindicated in patients receiving strong CYP3A4 inhibitors or inducers and in those with known hypersensitivity to the drug. Important warnings and precautions include neuroleptic malignant syndrome, tardive dyskinesia, metabolic changes, hyperprolactinemia, leukopenia/neutropenia, orthostatic hypotension, falls, seizures, and potential cognitive or motor impairment. Common adverse reactions in pediatric populations include nausea, somnolence, and extrapyramidal symptoms, including akathisia [9]. A randomized placebo-controlled trial in autistic disorder evaluated fixed doses of 20 mg/day and 60 mg/day and did not demonstrate a statistically significant advantage over placebo on the primary irritability outcome [10]. A 2025 Cochrane network meta-analysis of atypical antipsychotics for ASD reached a consistent conclusion, finding that lurasidone probably has little to no effect on irritability compared with placebo, in contrast to risperidone and aripiprazole [11]. Nevertheless, small case series have described potential benefit in selected patients, underscoring the need to report carefully characterized individual clinical experiences without exaggerating efficacy [12].

This report presents the longitudinal course of a Latin American child whose ASD was recognized only after extended psychiatric follow-up, despite longstanding developmental features that became apparent through retrospective history and structured clinical reassessment using Diagnostic and Statistical Manual of Mental Disorders, Fifth Edition, Text Revision (DSM-5-TR) diagnostic criteria [1]. Severe behavioral dysregulation, obsessive-compulsive symptoms, hallucinatory experiences, and intolerance to risperidone and aripiprazole further complicated treatment. This case demonstrates the diagnostic value of repeated neurodevelopmental assessments and the cautious, individualized use of low-dose lurasidone when standard pharmacologic options are not tolerated.

## Case presentation

A nine-year-old Latin American boy was brought by his mother to an outpatient child and adolescent psychiatric service in a Central American country for excessive irritability, frequent temper outbursts, difficulty adapting to school, and behavioral deterioration following the return to on-site classes after the coronavirus disease 2019 (COVID-19) pandemic. A detailed retrospective developmental and psychosocial history was obtained from his mother. He had no history of major or chronic medical conditions, surgical procedures, or previous hospitalizations, and the family medical and psychiatric history was noncontributory. Before the COVID-19 pandemic, he was described as a relatively socially withdrawn child who had difficulty establishing peer relationships and maintained only a few friendships during his school years. Retrospective developmental history also identified longstanding difficulties with reciprocal social interaction, rigid routines, restricted interests, and sensory sensitivities. Following the return to in-person schooling, his mother and teacher observed increasing irritability, frequent temper outbursts, difficulty adapting to school, inattentiveness, distractibility, impulsivity, and progressive behavioral dysregulation, representing a clear deterioration from his previous behavioral baseline. He described feeling persistently pressured and reprimanded at school, and a teacher report documented inattentiveness, distractibility, and impulsivity. There was no clinical evidence that the patient's symptoms were better explained by intellectual disability or global developmental delay. At the initial consultation, the presentation was conceptualized primarily as a mood- and stress-related disorder. Moderate depressive episode (ICD-10 F32.1) and adjustment disorder (ICD-10 F43.2) were recorded as working diagnoses, and fluoxetine 10 mg once daily was initiated.

Six weeks later, the mother reported marked clinical worsening, including increased anxiety, tremulousness, frequent crying, low mood, prolonged sleep-onset latency, and episodic symmetric clonic-appearing movements lasting several minutes and consistently occurring in emotionally stressful contexts. An electroencephalogram performed at an outside pediatric neurology facility was unremarkable, and no epileptic diagnosis was established; the episodes were considered possible functional neurological events, although none was captured on video-electroencephalography. Fluoxetine was discontinued, and sertraline 25 mg once daily and hydroxyzine 25 mg nightly were initiated. The motor episodes subsequently came under clinical control and were no longer a predominant concern.

During subsequent follow-up, sertraline was progressively titrated to a maximum dose of 150 mg once daily, with partial improvement in depressive and anxiety symptoms but persistent irritability and behavioral disturbance. Hallucinatory symptoms emerged after dose escalation. Although treatment-emergent hallucinations have rarely been reported with selective serotonin reuptake inhibitors (SSRIs) in children, available pediatric observational evidence does not show a clear association between antidepressant exposure and new-onset psychotic symptoms. Given the temporal relationship but insufficient evidence of causality, sertraline was continued with clinical monitoring.

By approximately month 13, the patient developed a distressing critical auditory voice alongside worsening behavioral symptoms, prompting the addition of risperidone 0.5 mg once daily. Risperidone produced only a partial clinical response, with persistent behavioral symptoms, and was discontinued several months later after the family reported a weight increase of approximately 4-5 kg during treatment.

Aripiprazole 3 mg twice daily was subsequently prescribed. Within the first weeks of treatment, the patient developed persistent nausea, vomiting, and dizziness that the family considered intolerable, and aripiprazole was discontinued. Five months later, the patient returned with escalating anxiety, persistent insomnia, severe irritability, aggressive outbursts, self-injurious behavior, and renewed perceptual symptoms, including a critical auditory voice and brief visual apparitions or figures in the peripheral visual field while fully awake. These experiences were recurrent and distressing and were reported by the family to cluster during periods of heightened stress, although occurrences outside these periods could not be excluded. The clinical record did not document the duration of individual episodes, the degree of insight retained during them, or a quantified frequency, and a dedicated structured psychosis-focused assessment was not performed at this stage.

Recognition of ASD reframed the patient's behavioral and perceptual symptoms within a neurodevelopmental context rather than a primary mood or psychotic disorder and directly informed the subsequent pharmacologic strategy. Because risperidone had been discontinued after clinically significant weight gain and aripiprazole after gastrointestinal intolerance and dizziness, an alternative antipsychotic was considered to target severe irritability, aggression, self-injurious behavior, insomnia, and persistent distressing hallucinatory symptoms. At the month-27 follow-up visit, off-label lurasidone 20 mg nightly with food was initiated, while sertraline 150 mg once daily was continued. Hydroxyzine had previously been discontinued and was not restarted. The patient's recorded weight at lurasidone initiation was approximately 86 kg. Longitudinal changes in weight and body mass index during treatment are shown in Figure 1.

**Figure 1:**
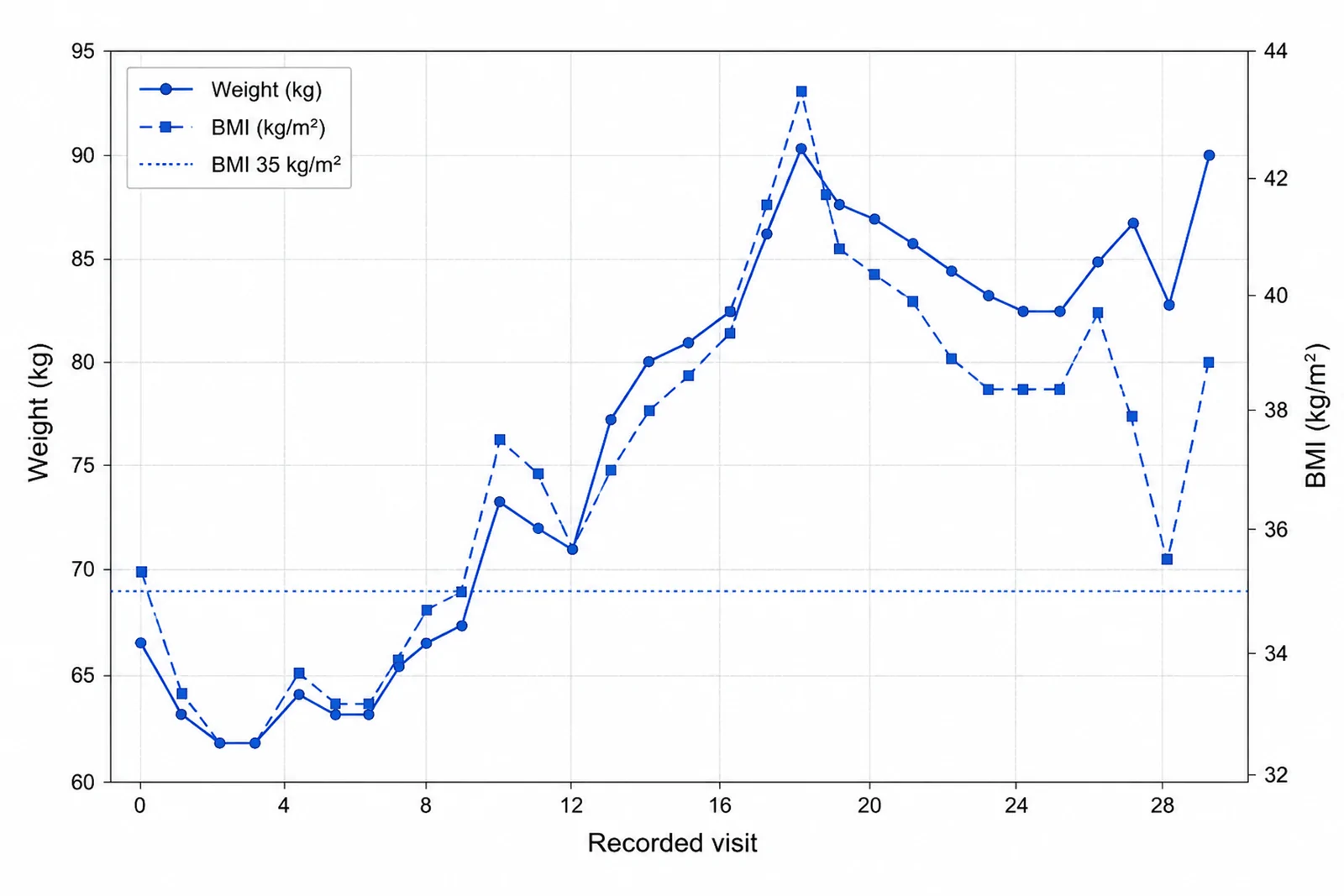
Longitudinal changes in weight and BMI during treatment Longitudinal changes in weight and BMI across recorded clinical visits. Weight increased from 66 kg at the first recorded visit to approximately 90 kg at the last recorded visit, with substantial fluctuations throughout treatment. The greatest increase in weight and BMI occurred during the period of risperidone treatment. At lurasidone initiation, the patient's recorded weight was approximately 86 kg. Subsequent weight measurements fluctuated during follow-up, with a final recorded weight of approximately 90 kg. Given the patient's age and the absence of complete height trajectories and BMI-for-age percentile data, these observations do not permit attribution of the subsequent weight trajectory to lurasidone or conclusions regarding weight stability during treatment. BMI: body mass index

Over the subsequent 12 months, the patient's family reported progressive improvement in irritability, with a substantial reduction in the frequency and severity of aggressive and self-injurious episodes. Sleep and anxiety also improved. Auditory and visual perceptual experiences were no longer reported by approximately the third month after lurasidone initiation and did not recur during the remaining observation period. This behavioral stabilization was accompanied by greater participation in family and educational activities, with caregivers reporting fewer aggressive episodes overall. No clinically apparent dystonia, parkinsonism, or other extrapyramidal symptoms were documented during follow-up, and the family reported no new prolactin-related symptoms. At the 12-month follow-up after lurasidone initiation, improvement in irritability, aggression, self-injurious behavior, anxiety, and sleep was maintained, with no recurrence of the previously reported perceptual symptoms. Lurasidone 20 mg nightly with food was continued together with sertraline 150 mg once daily and adjunctive behavioral interventions. The longitudinal clinical course, diagnostic evolution, pharmacologic interventions, and treatment outcomes are summarized in Table 1.

**Table 1:** Clinical course, diagnostic evolution, and pharmacologic management ASD: autism spectrum disorder; OCD: obsessive-compulsive disorder; EEG: electroencephalogram; DSM-5-TR: Diagnostic and Statistical Manual of Mental Disorders, Fifth Edition, Text Revision

Timepoint	Key clinical features	Diagnostic formulation	Pharmacologic intervention	Clinical course/outcome
Initial consultation (age 9 years)	Irritability, temper outbursts, school maladaptation, inattentiveness, impulsivity, and mood symptoms	Moderate depressive episode and adjustment disorder were considered working diagnoses based on prominent depressive symptoms, irritability, school maladaptation, and behavioral deterioration	Fluoxetine 10 mg/day	Mood- and stress-related formulation initially favored
Week 6	Anxiety, tremulousness, stress-associated clonic-appearing events, crying, and insomnia; external EEG unremarkable, with no epileptic diagnosis established	Depressive disorder; possible functional neurological events	Fluoxetine discontinued; sertraline 25 mg/day and hydroxyzine 25 mg nightly initiated	Fluoxetine discontinued after clinical worsening; events were considered possibly functional and subsequently came under clinical control
Month 3	Partial mood improvement with persistent irritability and behavioral dysregulation	Mood- and anxiety-related formulation remained predominant because depressive and anxiety symptoms partially improved, while irritability and behavioral dysregulation persisted	Sertraline was progressively titrated to a maximum of 150 mg/day. Hydroxyzine 25 mg was discontinued	Partial improvement in mood and anxiety; behavioral symptoms persisted. Higher-dose sertraline exposure preceded later hallucinatory symptoms, although causation was not established
~Month 13	Distressing critical auditory experiences and worsening behavior	The emergence of distressing auditory perceptual experiences and worsening behavior broadened the diagnostic formulation; however, the available clinical documentation was insufficient to establish a primary psychotic disorder	Risperidone 0.5 mg/day added	Partial response; subsequently discontinued after ~4-5 kg weight gain
~Month 22	Persistent complex behavioral and neuropsychiatric symptoms	Persistent multidomain behavioral and psychiatric symptoms despite sequential treatment prompted the reconsideration of the initial formulation and a more detailed neurodevelopmental reassessment	Aripiprazole 3 mg twice daily	Discontinued because of nausea, vomiting, and dizziness
Month 27	Anxiety, insomnia, severe irritability, aggression, self-injury, and auditory and visual hallucinatory experiences	ASD was diagnosed clinically according to DSM-5-TR criteria based on longstanding social communication difficulties and restricted/repetitive patterns of behavior, including rigid routines, restricted interests, and sensory sensitivities present since early childhood and associated with functional impairment. OCD was diagnosed based on distressing, ego-dystonic obsessive thoughts with repetitive handwashing and checking behaviors performed to neutralize intrusive thoughts	Lurasidone 20 mg nightly with food; sertraline continued	Progressive multidomain improvement over 12 months; hallucinatory symptoms absent by ~3 months; no adverse effects reported during lurasidone treatment

## Discussion

This case demonstrates two principal clinical messages that distinguish it from most prior reports. First, a longitudinal neurodevelopmental formulation may be required when a child presents with mood, anxiety, behavioral, or perceptual symptoms rather than through a direct referral for ASD assessment. Second, off-label alternatives may be considered with caution and explicit acknowledgment of the limited evidence base when severe irritability and behavioral dysregulation persist despite intolerance to both approved agents. The combination of delayed ASD recognition, prominent psychiatric comorbidity, and intolerance to both approved agents provides a clinically informative context.

Diagnostic complexity and delayed recognition of ASD

Irritability, school maladaptation, mood symptoms, anxiety, and stress-related complaints dominated the initial presentation. These features were clinically significant but nonspecific and initially directed attention toward mood and adjustment disorders. The eventual ASD diagnosis did not arise from a single late-onset symptom; rather, it followed a longitudinal reassessment in which retrospective developmental history revealed social communication difficulties, inflexible routines, sensory sensitivities, and restricted interests dating back to early childhood, assessed according to DSM-5-TR criteria by detailed clinical interview and direct behavioral observation rather than a standardized autism-specific rating scale. Thus, the delayed diagnosis is better understood as diagnostic masking by prominent psychiatric comorbidity and an evolving clinical picture rather than the new emergence of autistic features at age 9 [1,2].

The temporal association with return to on-site schooling may also have contributed to diagnostic anchoring on adjustment and mood disorders. Environmental transition can expose previously compensated social, sensory, and executive function vulnerabilities. A child may therefore appear to have developed a new psychiatric disorder when the transition has instead amplified longstanding neurodevelopmental difficulties. The case supports repeated developmental history-taking when symptoms are persistent, multidomain, or poorly explained by the initial formulation [2].

Interpreting hallucinatory symptoms in ASD

The reported perceptual symptoms resolved within approximately three months of initiating low-dose lurasidone (20 mg nightly) and did not recur during the 12-month follow-up period. However, treatment response and subsequent symptom resolution cannot reliably distinguish childhood-onset schizophrenia from perceptual or psychotic-like phenomena occurring in ASD. More importantly, the available longitudinal record did not establish the broader syndrome required for childhood-onset schizophrenia, including persistent psychotic symptoms accompanied by the characteristic clinical and functional features. Furthermore, although these symptoms emerged after escalating sertraline to 150 mg/day, the antidepressant was successfully maintained throughout the treatment period without symptom recurrence once clinical stabilization was achieved. The temporal association of these perceptual experiences with periods of heightened stress raised the possibility of a stress-related or neurodevelopmentally influenced phenomenon. However, because episode duration, quantified frequency, degree of retained insight, and findings from a structured psychosis assessment were not documented, the available data do not permit confident classification of these experiences as psychotic, stress-related, neurodevelopmentally mediated, or medication-associated phenomena [4,5].

Why lurasidone was selected

Risperidone and aripiprazole have the strongest regulatory and trial evidence for severe irritability associated with autistic disorder, but tolerability is highly individualized [6,7]. In this patient, risperidone was associated with a family-reported weight increase of approximately 4-5 kg, while aripiprazole was followed by nausea, vomiting, and dizziness severe enough to prompt discontinuation. The treatment team therefore faced a practical gap: the child continued to have severe behavioral dysregulation, aggression, self-injury, insomnia, and hallucinatory symptoms, but the two approved ASD-associated irritability options had not been tolerated [6,7].

Lurasidone was selected because antipsychotic coverage remained clinically necessary and because its receptor and tolerability profile differed from the previous agents. Lurasidone has high affinity for D2 and 5-HT2A receptors and also acts at 5-HT7 and 5-HT1A receptors [8]. These pharmacologic properties provide a mechanistic rationale for antipsychotic activity and may modulate mood and cognitive domains; however, receptor pharmacology alone should not be interpreted as proof of efficacy for ASD or OCD. In particular, claims that 5-HT7 antagonism is "procognitive" in ASD or that 5-HT1A partial agonism directly treats OCD would exceed the clinical evidence available from this case [8].

The 20 mg/day dose deserves attention. The randomized ASD irritability trial by Loebel and colleagues studied fixed doses of 20 mg/day and 60 mg/day, not 100-350 mg/day, and did not meet the primary endpoint on the Aberrant Behavior Checklist-Irritability subscale [10]. The negative trial is important and should not be minimized. Accordingly, this case should not be interpreted as evidence that lurasidone is generally effective for ASD-associated irritability. Instead, it documents an individual response in a clinically complex patient whose symptom targets exceeded irritability and included persistent hallucinatory experiences [10,11].

Observed response and alternative explanations

The temporal pattern was notable: multidomain improvement emerged after lurasidone initiation, and the hallucinatory symptoms were no longer reported by approximately three months. Behavioral improvement was sustained during 12 months of follow-up. Nevertheless, a causal attribution to lurasidone is not possible. Sertraline was continued; symptoms may have fluctuated spontaneously; the revised ASD/OCD formulation may have led to changes in family responses and environmental management; and the outcome was based on routine clinical assessment rather than blinded or standardized measures [10-12].

The absence of significant new weight gain was particularly relevant after the risperidone experience. Comparative pediatric antipsychotic data demonstrate clear between-drug heterogeneity in metabolic and prolactin effects [13]. However, because comprehensive longitudinal metabolic laboratory monitoring, including paired fasting lipids, fasting glucose, HbA1c, and serum prolactin, was not performed, objective assessment of metabolic safety is limited. It is therefore more accurate to state that no clinically apparent major adverse effects were documented than to conclude that lurasidone was metabolically neutral in this patient [13].

Relation to the existing literature

The existing evidence for lurasidone in ASD is sparse. The placebo-controlled trial of lurasidone for irritability in autistic disorder did not show significant efficacy at its primary endpoint at either 20 mg/day or 60 mg/day [10], and a 2025 Cochrane network meta-analysis reached the same conclusion at a higher level of synthesized evidence [11]. In contrast, Gupta and Hoover described four children and adolescents with ASD treated with lurasidone in a case series and reported improvement in irritability and mood-related symptoms in selected patients [12]. These case-based observations cannot overcome a negative randomized trial and a converging meta-analysis, but they may identify clinical phenotypes or treatment circumstances that warrant future study [10-12].

The current case differs from a straightforward ASD irritability presentation because treatment was pursued after intolerance to both approved agents and because the child also had OCD, anxiety, insomnia, self-injurious behavior, and distressing multimodal hallucinatory symptoms. The case therefore contributes primarily as a description of tailored clinical decision-making and longitudinal tolerability rather than as evidence of lurasidone efficacy for ASD as a diagnostic category [12].

Limitations

Several limitations of this case report must be acknowledged. First, as a single-patient observation, these data cannot establish the general efficacy, comparative safety, or direct causality of lurasidone in pediatric patients with ASD and comorbid psychiatric symptoms. Second, a child psychiatrist established the ASD diagnosis clinically using DSM-5-TR criteria [1], rather than standardized diagnostic instruments such as the Autism Diagnostic Observation Schedule, Second Edition (ADOS-2), Autism Diagnostic Interview-Revised (ADI-R), Childhood Autism Rating Scale, Second Edition (CARS-2), or Gilliam Autism Rating Scale, Second Edition (GARS-2) [14-17]. Similarly, comorbid OCD severity was not quantified using formal scales (e.g., Children's Yale-Brown Obsessive Compulsive Scale (CY-BOCS)), and the patient's hallucinatory experiences were not characterized using a structured psychosis interview. Although this reliance on clinical assessment and direct behavioral observation is a limitation, it mirrors real-world psychiatric practice in resource-constrained Latin American settings rather than representing a methodological shortcut. Third, clinical outcomes were determined based on clinician and caregiver reports rather than blinded, standardized rating scales such as the Aberrant Behavior Checklist-Irritability subscale or Clinical Global Impressions scales [18,19], which introduces potential reporting and expectation bias. Finally, the absence of complete longitudinal metabolic laboratory data including paired fasting lipids, glucose, HbA1c, and prolactin measurements restricts our assessment of metabolic safety to a descriptive account of clinical tolerability rather than objective laboratory verification. Despite these limitations, this case highlights the clinical importance of reconsidering the neurodevelopmental formulation when persistent multidomain psychiatric symptoms are not adequately explained by the initial diagnosis or do not respond as expected to treatment. The observed clinical course with lurasidone should be regarded as hypothesis-generating rather than evidence of efficacy. Future prospective studies should evaluate lurasidone in well-characterized pediatric ASD populations using standardized measures of irritability and global clinical improvement, systematic assessment of adverse effects and metabolic parameters, and longer-term follow-up. Controlled studies are needed to better define its potential efficacy, safety, and role, if any, in patients who cannot tolerate currently approved pharmacologic options.

## Conclusions

This case highlights the importance of reconsidering developmental history in children with persistent, multidomain psychiatric symptoms, as prominent anxiety, obsessive-compulsive symptoms, behavioral dysregulation, and atypical hallucinatory experiences may obscure an underlying ASD and contribute to diagnostic anchoring. Recognition of the neurodevelopmental context reframed the patient's clinical presentation and informed subsequent management. In this patient, intolerance to risperidone and aripiprazole limited standard pharmacologic options. Low-dose off-label lurasidone (20 mg nightly) was temporally associated with sustained improvement in irritability, aggression, self-injurious behavior, anxiety, sleep disturbance, and hallucinatory symptoms, with no clinically apparent major adverse effects documented during follow-up. However, this single case cannot establish causality or efficacy, particularly given the negative randomized evidence for lurasidone in ASD-associated irritability. Repeated neurodevelopmental reassessment, avoidance of diagnostic anchoring, and individualized treatment remain important when the initial diagnostic formulation or standard therapies do not adequately explain or control the clinical presentation.
